# Potential Uses of Olive Oil Secoiridoids for the Prevention and Treatment of Cancer: A Narrative Review of Preclinical Studies

**DOI:** 10.3390/ijms22031234

**Published:** 2021-01-27

**Authors:** Maria Rita Emma, Giuseppa Augello, Vita Di Stefano, Antonina Azzolina, Lydia Giannitrapani, Giuseppe Montalto, Melchiorre Cervello, Antonella Cusimano

**Affiliations:** 1 Institute for Biomedical Research and Innovation, National Research Council (CNR), 90146 Palermo, Italy; mariarita.emma@irib.cnr.it (M.R.E.); giuseppa.augello@irib.cnr.it (G.A.); antonina.azzolina@irib.cnr.it (A.A.); lydiagiannitp@gmail.com (L.G.); giuseppe.montalto@unipa.it (G.M.); 2 Department of Biological, Chemical, and Pharmaceutical Science and Technology (STEBICEF), University of Palermo, 90133 Palermo, Italy; vita.distefano@unipa.it; 3 Department of Health Promotion Sciences Maternal and Infantile Care, Internal Medicine and Medical Specialties, University of Palermo, 90133 Palermo, Italy

**Keywords:** secoiridoids, oleocanthal, oleacein, oleuropein, ligstroside, cancer

## Abstract

The Mediterranean diet (MD) is a combination of foods mainly rich in antioxidants and anti-inflammatory nutrients that have been shown to have many health-enhancing effects. Extra-virgin olive oil (EVOO) is an important component of the MD. The importance of EVOO can be attributed to phenolic compounds, represented by phenolic alcohols, hydroxytyrosol, and tyrosol, and to secoiridoids, which include oleocanthal, oleacein, oleuropein, and ligstroside (along with the aglycone and glycosidic derivatives of the latter two). Each secoiridoid has been studied and characterized, and their effects on human health have been documented by several studies. Secoiridoids have antioxidant, anti-inflammatory, and anti-proliferative properties and, therefore, exhibit anti-cancer activity. This review summarizes the most recent findings regarding the pharmacological properties, molecular targets, and action mechanisms of secoiridoids, focusing attention on their preventive and anti-cancer activities. It provides a critical analysis of preclinical, in vitro and in vivo, studies of these natural bioactive compounds used as agents against various human cancers. The prospects for their possible use in human cancer prevention and treatment is also discussed.

## 1. Introduction

Cancer incidence and mortality are rapidly growing worldwide, although cancer’s prominence as a cause of premature death is related to national levels of social and economic development. In Western developed countries, the increased number of new cancers is related to exposure to unhealthy lifestyles and environmental risks [1]. Risky behaviors, such as cigarette smoking, high fat diets, little physical exercise, and lack of daily fruit and vegetable consumption are also recognized cancer risk factors [2].

Diet and eating habits are essential in cancer prevention for two main reasons: first, abnormalities in food behavior and an unbalanced diet like the Western diet, are causes of several diseases; second, many phytochemical compounds contained in food, often in fruits and vegetables, protect against some diseases. Diet can play a dual role, like a two-faced Janus, as it can be a cause of or a protective factor against cancer at the same time. The Mediterranean diet (MD) is considered one of the healthiest worldwide dietary patterns due to a combination of foods rich in antioxidants and anti-inflammatory nutrients, such as fresh fruits and vegetables, and extra-virgin olive oil (EVOO), which play a protective role in cancer onset and which therefore may remarkably reduce the risk of cancer [3,4,5]. As supported by numerous epidemiological studies, the population of Mediterranean countries which traditionally follow this pattern of eating have a lower risk of chronic inflammation-associated diseases, including cancer [6].

EVOO is a main component of the MD. Several epidemiological as well as preclinical studies support the health benefits and the importance of EVOO in cancer prevention, mainly related to its antioxidant power [7,8]. More recently, it has been hypothesized that the cancer preventive capacity of olive oil may be mediated, at least in part, by the presence of minor components (about 2% of oil weight) which include more than 230 chemical compounds [9,10]. In particular, the beneficial effects of EVOO have been attributed to phenolic compounds, such as phenolic alcohols, hydroxytyrosol (3,4-dihydroxyphenylethanol; 3,4-DHPEA), and tyrosol (p-hydroxyphenylethanol; p-HPEA), along with their secoiridoid derivatives 3,4-DHPEA-EA (oleuropein aglycone), p-HPEA-EA (ligstroside aglycone), 3,4-DHPEA-EDA (oleacein, OA), p-HPEA-EDA (oleocanthal, OC), and oleuropein [10]. All these components have been isolated, and their biological activities investigated, addressing the definition of pharmacological properties, molecular targets, and action mechanisms. A considerable amount of evidence contributes to defining the wide spectrum of biological effects of these compounds, which includes cardioprotective, antimicrobial, neuroprotective, and anti-cancer effects [11]. It has been reported that these compounds may improve antioxidant and anti-inflammatory protection in the context of different disorders through the modulation of various molecular pathways [12].

Inflammation is the immune system’s response to infection and injury, and it is the first step in the development of many diseases, such as arthritis, cancer, and stroke, as well as neurodegenerative and cardiovascular diseases. The anti-inflammatory activity of the phenolic components of EVOO has been investigated in many inflammation-related diseases [13]. For example, it has been reported that OC targeted different inflammation mediators, such as COXs [14] and inducible nitric oxide synthase (iNOS) [15], whereas in an Alzheimer’s disease model, OC protected neurons by inhibiting tau fibrillation [16]. In addition, OC also protected hippocampal neurons from Aβ-derived diffusible ligand (ADDL) toxicity [17] and reduced the inflammation activation of astrocytes in the hippocampus [18]. Similar evidence has been published for other phenolic components [10,13,19].

Since chronic inflammation is considered a promoting factor in the early stages of carcinogenesis, in recent years attention has been turned to the anti-cancer properties of EVOO phenols.

This review aims to summarize the most recent findings regarding the preventive and anti-cancer activity of secoiridoids, providing a critical analysis of preclinical, in vitro and in vivo, studies in which these natural bioactive compounds are used as agents against various human cancers.

## 2. Chemistry of Secoiridoids

**Figure 1:**
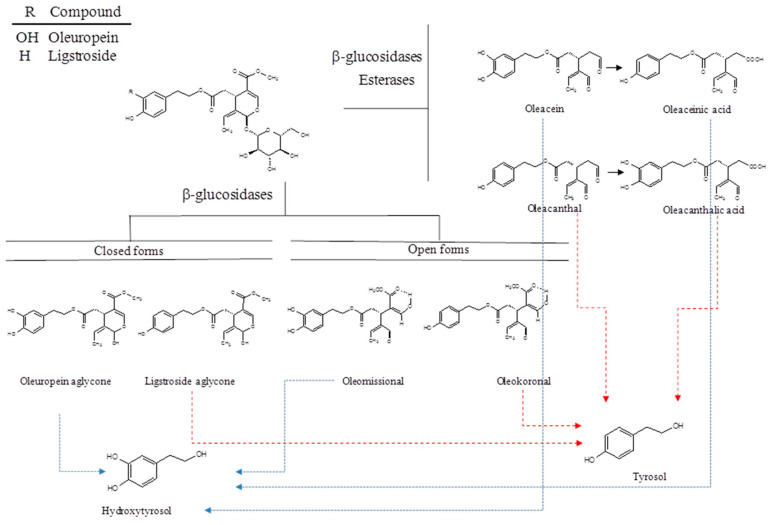
Chemical structures of phenolic alcohols and secoiridoids compounds in EVOO.

Secoiridoids are a group of compounds found exclusively in all 500 species of Oleaceae plants, including the European olive tree (*Olea europaea* L.), and they comprise the majority of bioactive polyphenols in olive oil and drupes [10,20,21,22,23,24]. Most secoiridoid phenolic derivatives in olive oil come from oleuropein and ligstroside, which are the major secoiridoids in the olive fruit (Figure 1).

During crushing and malaxation for the production of olive oil, oleuropein and ligstroside come into contact with β-glucosidase and are transformed into the corresponding oleuropein and ligstroside aglycones (p-HPEA and 3,4-DHPEA, respectively). These two forms are unstable in olive oil; indeed, in a non-aqueous medium, oleuropein and ligstroside aglycones do not exist and are mainly transformed into the more stable dialdehyde form of decarboxymethyl elenolic acid esterified with tyrosol and hydroxytyrosol (also named oleocanthal or p-HPEA-EDA, and oleacein or 3,4-DHPEA-EDA).

EVOO can also contain other derivatives, such as the stable enolic form of oleuropein and ligstroside and aglycones, named oleomissional and oleokoronal, whose structures have recently been completely elucidated by Diamantakos et al. using nuclear magnetic resonance [25]. Oxidation products of oleocanthal (OC) and oleacein (OA) were found in fresh oils in very low concentrations. The concentration of oleaceinic acid and oleocanthalic acid increased with storage time, while the oleacein and oleocanthal concentration decreased [26].

## 3. Extraction of Phenolic Alcohols and Secoiridoids

In the literature, there are numerous methods used for purifying olive oil phenolic constituents. Montedoro and collaborators were the first to study the secoiridoid class in 1993; they are responsible for the structural characterization of ligstroside and oleuropein aglycones from virgin olive oils [27].

OC was later identified by Unilever Research and Development, Vlaardingen (Netherlands) [28]. In particular, the published methods relating to the isolation of OC provide for its extraction from the enriched polyphenolic fraction. The subsequent extraction of the total polyphenolic fraction, using hexane or polar solvents such as methanol and aqueous ethanol and acetonitrile, allowed the isolation and characterization of OC [14,20,21,22,23,24,25,26,27,28,29,30]. Other isolation methods require the use of preparative HPLC and reversed-phase stationary phases or even sophisticated technologies, such as high-performance counter-current chromatography (HPCCC) [31]. A pioneering annular centrifugal extractor (ACE)-based extraction has recently been developed for a pilot-scale laboratory, leading to the recovery of the biophenol-enriched fraction from olive oil for the first time.

This highly productive method, with the advantage of industrial scale potential, provides huge amounts of high value-added compounds, such as oleocanthal, in their pure forms in a short period of time. This method allows the isolation of biophenols in quantity to meet the needs of in vivo experiments [32].

Given the important biological activities of oleocanthal, some synthetic approaches have been tried in order to obtain quantities of the compound useful for biological studies. The first attempt at a complete synthesis of both OC enantiomers was published by Smith II and collaborators in 2005 [33]. Other synthesis techniques have been described in the literature, though they have numerous steps and very low yields [34,35,36].

A second-generation synthetic sequence was used to obtain quantities on the order of multigrams. In addition, a series of OC analogs useful for future experiments in the biological field have been synthesized. The most interesting production path is that of Valli et al. (2013) in which oleocanthal is prepared in just eight steps with an approximate average yield of 9%. This approach is the shortest route published to date. Furthermore, both biologically active enantiomers of OC can be separated by the efficient resolution of the racemic mixture on an enantioselective HPLC column [37]. Finally, a recent strategy involves the large-scale synthesis of OC and analogues starting from oleuropein obtained from olive leaves [38].

Another possible way to obtain phenolic extracts for use as natural food antioxidants, food supplements, pharmaceutical solutions, or ingredients in functional foods, involves the use of olive oil mill waste (OOMW). Hydroxytyrosol, known for its interesting pharmacological activities and its antioxidant activity, is one of the major phenolic compounds present in OOMW. Unfortunately, its use has been limited, until recently, because it is not a commercially available compound.

Numerous methods have been studied for the purification of hydroxytyrosol from the by-products of olive processing; some patents have used counter-current liquid-liquid extraction [39], adsorbent resins [40], supercritical fluid extraction with a column operating in counter-current mode [41] and, finally, adsorption in non-ionic resins [42]. The method considered most efficient to date is certainly that of Fernández-Bolaños which, in a simple, practical and economical way, allows the extraction of highly purified (up 90%) hydroxytyrosol from the by-products of OOMW [43].

A number of methods have been proposed for the extraction of phenolic compounds from olive leaves. The most commonly used techniques involve conventional solid-liquid extraction and ultrasonic extraction [41]. Recently, a number of non-conventional technologies have been proposed for the extraction of oleuropein [44], including separation by membrane, infrared-assisted methods, accelerated solvent extraction, extraction with supercritical fluids [45], acid hydrolysis by sulfuric acid and microchannel devices [44,45,46,47,48,49,50,51].

## 4. Oleocanthal

In 1993, Montedoro et al. discovered and revealed the chemical structure of a phenolic component of olive oils, later named oleocanthal (OC) [27], which is also called decarboxymethyl ligstroside aglycone [52], a dialdehydic form of deacetoxy-ligstroside aglycone [34], a dialdehydic form of deacetoxyligstroside glycoside [53], deacetoxy-dialdehydic ligstroside aglycone [54], deacetoxy ligstroside aglycone [55], and p-hydroxyphenylethanol-elenolic acid dialdehyde [56]. The International Union of Pure and Applied Chemistry (IUPAC) name for OC is 2-(4-hydroxyphenyl)ethyl(3S,4E)-4-formyl-3-(2-oxoethyl)hex-4-enoate, with the Chemistry Abstracts Service (CAS) number 289030-99-5 [57].

In 2005, Beauchamp et al. identified in OC the pungent component of EVOO that induces a strong prickling sensation in the throat, similar to that caused by the non-steroidal anti-inflammatory drug (NSAID) ibuprofen [14]. NSAIDs such as ibuprofen are COX inhibitors because they suppress cyclooxygenase (COX) enzyme activity. COXs regulate the biosynthesis of prostaglandins, important mediators of inflammation, from arachidonic acid. During inflammatory response, prostaglandin synthesis and COX expression levels are significantly increased.

In their study, Beauchamp et al. showed that OC acts like a non-selective COX inhibitor, ibuprofen-like, and an anti-inflammatory agent [14]. The 50% inhibition (IC50) concentrations for (−)-oleocanthal are 23 µM and 28 µM for COX-1 and COX-2, respectively; IC50 values for (+) oleocanthal are 25 µM and 40 µM for COX-1 and COX-2, respectively; IC50 values for ibuprofen are 5 µM and 223 µM for COX-1 and COX-2, respectively. This feature of OC turned out to be very important as a property of EVOO that, combined with the antioxidant feature of other phenolic components, made olive oil an essential element of a healthy diet.

Together with its anti-inflammatory and antioxidant activity, the anti-cancer activity of OC has been demonstrated on several cancer models, both in vitro and in vivo. Recently, we have shown that OC exerts a potent anti-cancer activity against hepatocellular carcinoma (HCC) and colorectal carcinoma (CRC) cells [58]. In HT29 and SW480 colon cancer cells, 25–50 µM of OC inhibited colony formation and induced apoptosis by increasing ROS (reactive oxygen species) production that causes DNA damage and consequently cell death [58]. In HCC cells, OC treatment elicited the expression of the γH2AX marker for DNA damage, increased intracellular ROS production, and caused mitochondrial depolarization in a dose dependent manner, leading to cell death [58]. These results highlight different effects of phenol used at different doses as an anti- or pro-oxidant.

In the HT29 colon cancer cell model, OC activated adenosine monophosphate-activated protein kinase (AMPK) [59]. AMPK acts as a sensor of cellular energy status involved in cancer cell apoptosis [60]. In HT29 cells, OC suppressed the expression of COX-2 protein and activated AMPK, resulting in the inhibition of cell viability and proliferation, and inducting apoptosis. The knockdown of AMPK in HT29 cells attenuated the apoptosis induced by OC, suggesting that AMPK is a molecular target of OC. The same authors showed that OC exhibited a strong inhibitory effect on TPA-induced neoplastic cell transformation in JB6 Cl41 mouse epidermal cells by downregulating AP-1 activity through the inhibition of ERK1/2 phosphorylation [59]. An in vivo chicken chorioallantoic membrane (CAM) assay with HT29 cells confirmed a reduction of the tumoral areas after OC treatment [59].

Moreover, (−)-oleocanthal inhibited the growth of human breast (MCF-7, MDA-MB-231) and prostate (PC-3) cancer cell lines by inhibiting the phosphorylation of c-Met kinase [61]. c-Met is a tyrosine kinase receptor (RTK) that binds the ligand hepatocyte growth factor (HGF). The binding of HGF to c-Met causes receptor autophosphorylation that induces downstream signaling through several pathways (i.e., Rac1/Cdc42, PI3-kinase/AKT and ERK/mitogen-activated protein pathways) involved in cellular proliferation, motility, migration, and invasion [61,62]. The inhibitory effect of OC on the HGF-induced c-Met phosphorylation/activation caused a decrease in AKT and MAPK phosphorylation that resulted in an inhibition of HGF-induced cell proliferation, invasion, and G1/S cell cycle progression [63]. The dysregulation of the HGF/cMet pathway affects migration and invasion via the Brk/paxillin/Rac1 signaling pathway that is inhibited after treatment with OC. Moreover, this inhibitory effect is correlated with an inhibition of epithelial to mesenchymal transition (EMT), as highlighted by increased expression levels of epithelial markers, such as E-cadherin and Zo-1, and the decreased expression of the mesenchymal marker vimentin [63]. EMT is a developmental process that regulates the transition of cells from epithelial (E) to mesenchymal (M) phenotypes and that, in cancer progression, is related to invasion and metastasis [64]. Furthermore, correlated to its inhibitory effect on c-Met, OC showed anti-angiogenic activity via the downregulation of expression levels of the microvessel density marker CD31 in endothelial colony-forming cells [60]. OC treatment not only affected cells grown in monolayers, but it has been demonstrated that at a dose of 20 μM it inhibited the growth of HGF-induced 3D spheroids of human breast MDA-MB-231 cancer cells and human prostate DU145 cancer cells [65].

OC has also been used in combination with different drugs to improve the efficacy of cancer treatment. Ayoub et al. observed a synergistic growth inhibition of BT-474, MCF-7, and T-47D breast cancer cells treated with OC plus tamoxifen, as demonstrated by combination index values of 0.65, 0.61, and 0.53 for each cell line, respectively. Mechanistically, treatment with OC induced a decrease of estrogen receptor alpha (ERα) levels. To explain this result, the authors performed in silico docking studies that highlighted overlapping estrogen receptor binding modes of OC and 17β-estradiol, whereas the binding modes were different between OC and tamoxifen. Indeed, a combination with 17β-estradiol did not affect the activity of OC on cancer cells [66]. This study validated the in vitro and in vivo efficacy of OC in modulating ER expression and function in breast cancer cells, and its synergy with the selective ER modulator tamoxifen [66].

OC, as a c-Met inhibitor, has been combined with lapatinib (LP), a dual epidermal growth factor receptor (EGFR)/HER2 inhibitor approved by the US Food and Drug Administration (FDA). Treatments with OC/LP resulted in synergistic anti-proliferative effects in HER2-positive BT-474 and SK-BR-3 breast cancer cell lines [67]. In particular, in BT-474 cells, the effect was cytostatic, as demonstrated by an increase of cells in the G1 phase in cell cycle analysis, and in SK-BR-3 cells the effect was cytotoxic, with apoptosis induction. In addition, combined OC/LP treatment significantly suppressed the activation of c-Met, HER2, and EGFR in both cell lines and suppressed the activation of AKT in BT-474 cells. These data were also confirmed in vivo in an orthotopic xenograft tumor model of BT-474 cells in nude mice. After treatment with OC plus LP, a greater tumor growth inhibition was observed, compared to controls, vehicle and single drugs. Furthermore, data showed the inhibition of multiple downstream survival and mitogenic signaling pathways in HER2-positive cells, including the PI3K, MAPK, and STAT pathways [67]. The same nude mouse xenograft model generated by orthotopic inoculation with BT-474 cells has been used by Siddique et al. to investigate the effect of OC in breast cancer recurrence. Actually, despite progress in therapies for treatment of breast cancer and improvements in survival rates, poor results in recurrence prevention occurred [68,69]. The study of Siddique et al. showed that OC significantly suppressed the development of new tumors and inhibited local recurrence in 50% of treated animals. At the molecular level, OC treatment stabilized E-cadherin and reduced vimentin, decreased phosphorylation levels of both HER2 and MET in recurrent tumors, resulting in the inhibition of EMT and cell invasiveness [70].

c-Met and COX-2 are the targets of OC involved in the suppression of lung cancer progression and metastasis, as tested in vitro and in vivo using A549 cells in a nude mouse tail vein injection model [71].

Another action mechanism of OC in inhibiting cell proliferation and migration is by modulating Ca2+ ion levels. In MCF-7 and MDA-MB-231 breast cancer cells, OC impaired cell migration and inhibited cell proliferation, while it had no effect on MCF10A non-tumoral cells. OC stimulates Ca2+ influx via the downregulation of the transient receptor potential cation channel, subfamily C, member 6 (TRPC6), as confirmed by *TRPC6* expression silencing [72].

PI3K/AKT/mTOR is a relevant pathway involved in cell proliferation frequently altered in cancer. Moreover, AKT mediates numerous cellular functions including angiogenesis, metabolism, growth, proliferation, survival, protein synthesis, transcription, and apoptosis [73]. Khanfar et al. have demonstrated, by in vitro molecular docking experiments, that OC bound and inhibited mTOR kinase with an IC50 value of 708 nM. After treatment with OC, phosphorylated levels of mTOR decreased, resulting in an anti-proliferative effect on MCF-7, T47D and MDA-MB-231 breast cancer cells, as well as Caco colon cancer cells. The breast cancer cells were more sensitive, with lower IC50 values [74].

In PC3 prostate cancer cells, MDA-MB-231 breast cancer cells, and BxPC3 pancreatic cancer cells, OC induced cell death via lysosomal membrane permeabilization (LMP), whereas it induced only reversible cell cycle arrest in non-cancerous cells [75]. LMP is induced by different stimuli and is the cause of the release of lysosomal enzymes into the cytoplasm which, in turn, lead to cell death [76]. Low levels of LMP damage cells and trigger apoptosis, whereas high levels of LMP kills cells rapidly and directly by necrosis. After treating MCF-7 human breast cancer cells and PC3 prostate cancer cells with OC, the authors observed the translocation of galectin-3 and cathepsins into the cytosol, markers of damaged lysosomal membranes, also evidenced by the LysoTracker test [72]. Data were confirmed in vivo in a genetically engineered PNET, RIP-Tag mouse model of pancreatic cancer; OC increased survival and reduced tumor size via LMP [74]. Finally, treatment of PC3 and MDA-MB-231 cancer cells with an EVOO-OC-enriched medium confirmed that the antitumoral activity of EVOO was directly and linearly correlated to the oleocanthal content [77].

In the human multiple myeloma (MM) ARH-77 cell model line, OC inhibited cell proliferation, induced cell cycle arrest in G0/G1, and apoptosis by downregulating the ERK1/2 and AKT pathways and activating p38 kinase. Furthermore, OC reduced the expression and secretion of MIP-1α protein, leading to RANKL downregulation which is involved in the development of osteolytic bone lesions in MM [78].

OC exerts its antitumoral activity also against melanoma [79]. One of the most important signaling pathways involved in the progression of melanoma is the signal transducer and activator of the transcription 3 (STAT3) pathway [80]. In melanoma cells, OC suppressed proliferation, migration, invasion and induced apoptosis by downregulating Mcl-1, Bcl-xL, MMP-9, MMP-2 genes expression as well as the phosphorylation and activation of STAT3; it also suppressed the expression of JAK2 and Src kinases, inhibiting cell invasion and angiogenesis [79]. Similarly to MM, the inhibition of ERK1/2 and AKT phosphorylation and downregulation of Bcl-2 expression were the main mechanisms by which OC inhibited cell proliferation in melanoma cells when compared to normal dermal fibroblasts [79,81].

In histiocytic lymphoma U937 cells, OC acts as an Hsp90 inhibitor as shown by the significant reduction of the expression levels of some Hsp90 client proteins (AKT and Cdk4) [79]. A direct non-covalent interaction between the Hsp90-ATP binding site and OC has been observed, resulting in the inhibition of ATPase activity of the chaperone. Moreover, OC induced cell cycle arrest in the G2/M phase and apoptosis in U937 cells, though it only had a slight effect on the viability of peripheral blood mononuclear cells (PBMC) [82].

In HCC cells, a study performed on HepG2, Huh7, and Hep3B HCC cells using a polyphenolic extract containing OC and ligstroside aglycone was shown to inhibit cell viability and increase cell death [83]. Treatment with phenolic combination resulted in an increased level of the phosphorylated form of AKT and ERK, associated with a slight increase in the LC3II/LC3I ratio and p62, suggesting the activation of autophagy. The addition of TNFα, a pro-inflammatory cytokine, potentiates the anti-proliferative effect of EVOO extract [83]. Furthermore, OC suppressed HCC cell migration, invasion, and metastasis both in vitro and in vivo in an orthotopic HCC model. OC acted by inhibiting IL-6-induced STAT3 activation, and Twist was consequently inhibited as well as EMT. Furthermore, OC modulated STAT3 levels acting on STAT3 positive regulators p-JAK1 and p-JAK2 and suppressing levels of SHP-1, a negative regulator of STAT3 [84]. Recently published evidence showed that OC inhibited neurite growth and prevented the growth and proliferation of the NB2a mouse neuroblastoma cell line by increasing oxidative stress and apoptosis [85].

All together, these studies (Table 1) highlight OC’s strong anti-cancer activity that results in the inhibition of proliferation, cell cycle arrest, and apoptosis induction. Alongside its better documented anti-inflammatory and antioxidant activity, its pro-oxidative action has also been reported, as shown by the dose-dependent ROS production and consequent cell damage and death. Furthermore, the inhibitory action of EMT observed in different models is also noteworthy.

## 5. Oleacein

Oleacein (OA) is derived from oleuropein by spontaneous chemical processes and is further converted into eleonolic acid and hydroxytyrosol [86]. More effective than oleuropein, OA inhibited cell proliferation, colony formation, and migration in A43 human epidermoid cancer cells used as a cutaneous non-melanoma skin cancer model. The molecular events that lead to growth inhibition are related to the shut-down of proliferative signals, such as decreased levels of B-Raf, p-AKT and p-ERK proteins after treatment with OA [87]. OA also inhibited cell growth in cells cultured as 3-D spheroids [88].

In SH-SY5Y human neuroblastoma cells, OA had anti-proliferative and anti-metastatic effects by blocking the cell cycle in the S phase, upregulating pro-apoptotic proteins Bax and p53, as well as decreasing the expression of the pro-survival protein Bcl-2 and STAT3 [89]. 

Despite the small number of studies conducted on a few cancer models, the data obtained so far show that OA has an anti-proliferative action related to the inhibition of proliferative signals and the upregulation of pro-apoptotic proteins (Table 1).

## 6. Tyrosol and Hydroxytyrosol

Tyrosol (Tyr; p-hydroxyphenylethanol) and hydroxytyrosol (HTyr; 3,4-dihydroxyphenylethanol) are two simple phenolic components of olive oil with well-established antioxidant and anti-inflammatory qualities. In general, these characteristics are attributable to their ability to chelate oxidizing agents, therefore acting as radical chelators [13].

Since chronic inflammation and alterations of normal cellular redox status are two of the main features that characterize and promote neoplastic transformation, Tyr and especially HTyr, as well as other olive oil polyphenolic compounds, have been widely used to evaluate their potential anti-cancer effects in the context of different human malignancies, including CRC, prostate and breast cancers, and HCC [90].

It has been reported that one of the main mechanisms by which HTyr may exert its antitumor effects is its autoxidation, with a consequent production and accumulation of H_2_O_2_ in the culture medium. Fabiani et al. demonstrated, in fact, that the anti-proliferative effects of HTyr are inversely correlated to the ability of different tumor cells to remove H_2_O_2_ from culture medium [91]. Therefore, tumor cell sensitivity to HTyr treatment may be modulated by adding compounds such as catalase or pyruvate, that do not favor H_2_O_2_ accumulation, to the culture medium [91].

However, it has been reported that HTyr may act as an anti-cancer agent by inducing the suppression of cell survival and/or activating pro-apoptotic pathways. Corona et al. demonstrated that HTyr treatment induced cell cycle arrest in colon cancer cells, with a significant reduction of the phosphorylation state of ERK1/2 as well as downstream cyclin D1 [92]. In addition, it has been reported that HTyr is able to induce apoptosis in DLD1 colon cancer cells though ROS production. ROS induced the activation of the PI3K/AKT/FOXO3 pathway with a consequent modulation of FOXO3 targets, such as SOD and catalase, which contributed to decreasing cellular antioxidant defences [93].

The reduced expression of EGFR is associated with a decrease of cell proliferation in colon cancer cells. The induction of ubiquitination and consequent lysosomal degradation of EGFR, both in vitro and in vivo, is another molecular mechanism that has been proposed to explain the anti-proliferative effect of HTyr treatment in colon cancer [94]. It has been shown, in fact, that HTyr treatment induced the phosphorylation of CBL, an E3 ubiquitin-protein ligase, which enhanced its ubiquitin-ligase activity leading to the ubiquitination and lysosomal degradation of EGFR. Pre-treatment with a proteosomal inhibitor, MG132, reversed all these effects. Therefore, interestingly, the authors reported that the co-treatment of tumor cells with both HTyr and Tyr produced synergistic effects reducing cell proliferation and EGFR expression [94].

In addition, investigations of HTyr’s effects in colon cancer proliferation both in vitro and in vivo suggested the ability of olive oil phenolic extracts to regulate epigenetic mechanisms. CpG methylation on the promoter of the Type I Cannabinoid Receptor (CB1), which may act as a tumor suppressor, has been often reported in the context of different malignancies, including colon cancer [95]. The administration of olive oil phenolic extracts, including oleuropein and HTyr, rescued the expression of the CB1 gene, reducing the methylation status of its promoter and simultaneously reducing tumor cell proliferation in vitro [96].

More recently, Hormozi et al. demonstrated that HTyr treatment of human CRC LS180 cells induced the expression of pro-apoptotic genes, such as *CASP3* and *Bax*, and increased antioxidant enzyme activity, leading to a reduction of tumor cell proliferation [97].

Recently, the ability of HTyr to modulate tumor cell antioxidant activities was also investigated in breast cancer cells, under hypoxic and normoxic conditions [98]. It has been shown that HTyr is able to affect cancer cell proliferation, mainly in hypotoxic conditions, modulating the transcription and translation of different proteins involved in peroxisome proliferator-activated receptor gamma coactivator 1-alpha/Nuclear factor erythroid 2-related factor 2 (PGC-1α/Nrf2) and peroxisome proliferator-activated receptor gamma coactivator 1-alpha/Estrogen-related receptor α (PGC-1α/ERRα) pathways, responsible for cellular antioxidant response [98]. Although it reduces ROS levels, the same authors showed that HTyr does not affect NO levels. The authors investigated the molecular mechanisms of HTyr in MCF7 cells under hypoxic conditions in more detail. HTyr in a dose dependent manner reduced the levels of the HIF-1α protein but not of the corresponding mRNA. Furthermore, at higher concentrations HTyr determines an up regulation of adrenomedullin (AM) and vascular endothelial growth factor (VEGF), and the authors concluded that HTyr acts via HIF-dependent and -independent regulatory mechanisms. In fact, at high doses HTyr acts as an aryl hydrocarbon receptor (AHR) ligand, inducing AM and VEGF genes expression. The authors suggested that at high doses HTyr may acts as an AHR agonist favoring the induction of angiogenic genes under hypoxic conditions [99]. These results highlighted how the action of the phenolic compounds can have opposite and controversial effects in different conditions and especially at different concentrations.

Recent advances in the study of the impact of HTyr in regulating breast cancer cell oxidative status showed that combinations of HTyr with common chemotherapeutic agents, such as paclitaxel, had synergistic effects in reducing tumor growth both in vitro and in vivo [100]. It is well-established that treatment with common taxanes, such as paclitaxel, may induce ROS production which has been identified as the major cause of cardiotoxic side effects of these drugs [101,102]. The addition of HTyr as a co-adjuvant in the treatment of breast cancer with paclitaxel ameliorated the high oxidative damage induced by chemotherapy and had significant synergistic effects in reducing tumor cell proliferation [100].

In addition, the effects of HTyr in regulating autophagy was recently documented in triple negative MDA-MB-231 and in ER positive MCF-7 breast cancer cells [102,103]. Autophagy is a well-conserved molecular mechanism whose role remains unclear and is often controversial in different human malignancies. In breast cancer, the inhibition of autophagy mediated by treatment with a common autophagy inhibitor, 3-methyladenine, increased tumor cell migration and invasion. On the contrary, treatment of cells with HTyr or oleuropein reversed 3-MA-dependent suppression of autophagic flux, increasing LC3II/LC3I and reducing p62 expression, leading to a reduction of tumor cell migration and invasion [103,104].

Recent evidence has shown that the inhibitory effect of HTyr in regulating breast cancer cell migration and invasion is dependent on its ability to target EMT, Wnt/β-catenin, and transforming growth factor-β (TGF-β) pathways [105]. HTyr treatment in different breast cancer cell lines decreased, in fact, β-catenin and cyclin D1 protein expression and reduced the expression of EMT markers such as Snail and vimentin. These effects were accompanied by the reduced phosphorylation of SMAD2/3 that was correlated to lower TGF-β activity [105]. Similar results were obtained in prostate cancer in which HTyr induced tumor cell growth arrest and apoptosis, regulating multiple molecular signaling pathways. Zubair et al. reported that HTyr treatment of prostate cancer cells affected cell proliferation, inhibiting the AKT, STAT3 and NF-κB pathways, and induced cell apoptosis, enhancing the expression of pro-apoptotic markers such as Bax and Bcl-2 [106]. Accordingly, Zhao et al. have previously demonstrated similar effects of HTyr treatment on the AKT and NF-κB pathways in HCC, both in vivo and in vitro, leading to a reduction of tumor cell prolife ration and angiogenesis [107].

Furthermore, it has been shown that HTyr exerted its anti-proliferative and pro-apoptotic effects in human HCC cells by inhibiting the expression of fatty acid synthase (FAS) and farnesyl diphosphate synthase (FPPS), whose higher expression was associated with higher aggressiveness of this cancer [108].

Unlike HTyr, Tyr is rarely employed as an anti-cancer agent and is found in more common therapeutic applications in non-malignant disorders, including diabetes, non-alcoholic fatty liver disease (NAFLD), and cardiovascular disorders [13]. Only in glioblastoma cells did Tyr show more efficient anti-cancer activities than HTyr and oleuropein by inhibiting TNF-α-induced COX2 expression and phosphorylation of JNK, ERK and NF-κB, leading to a significant reduction of tumor cell migration [109].

All together, these studies suggest that HTyr and, despite less evidence, Tyr may act as anti-cancer agents through the inhibition of cell proliferation and the induction of apoptosis (Table 2).

## 7. Oleuropein

Oleuropein and ligstroside, along with OC and OA, are the most abundant phenolic compounds found in EVOO. Oleuropein is an ester of HTyr.

Both oleuropein isoforms, its glycosidic and aglycone forms, have shown antioxidant, anti-inflammatory, and anti-cancer effects. Several studies have demonstrated that oleuropein inhibited cell growth and induced apoptosis in different cancer cell lines and had anti-cancer effects in animal studies [110,111].

Experimental evidence has proven that exposure to an olive leaf extract enriched in oleuropein reduced proliferation and motility in different cancer cells, such as melanoma [112], colon carcinoma [113], breast cancer [114] and chronic myeloid leukemia [115]. These effects are due to metabolic inhibitory activity exerted by oleuropein that neutralizes the aerobic glycolysis exploited by tumor cells, revealing that it may be effectively used as a complementary anti-cancer therapy [112].

In human colon cancer HT29 and SW620 cell lines, oleuropein glycoside treatment resulted in a significant inhibition of cell proliferation, cell cycle arrest, and apoptosis [113]. In another study, exposure of HT29 cells to oleuropein induced apoptosis in a p53-dependent manner and provoked a decrease in levels of hypoxia inducible factor-1 α (HIF-1α) protein expression [116]. Moreover, in in vivo studies of mice consuming a basal diet with oleuropein, it prevented azoxymethane (AOM)-induced colon cancer and, in addition, reduced DNA damage in peripheral leukocytes [117]. Similarly, the administration of oleuropein in mice co-exposed to dextran sulfate sodium (DSS) and AOM induced a decrease in inflammation markers, and a reduction in colon tumor development [118].

In vitro experiments also supported an anti-cancer effect of oleuropein in HCC [119]. HepG2 and Huh7 HCC cell lines treated with oleuropein showed an inhibition of cell proliferation and induction of apoptosis in a ROS-dependent manner. Moreover, oleuropein with cisplatin combination therapies showed antitumor activity in HCC cell lines by targeting the pro-nerve growth factor (pro-NGF)/NGF signaling pathway [120].

In SH-SY5Y neuroblastoma cells, oleuropein treatment has been shown to increase the expression levels of proteins related to cell proliferation, such the p53 gene and CDK inhibitors (CDKN1A, CDKN2A and CDKN2B) [121]. In thyroid cancer cell lines, oleuropein treatment reduced the levels of AKT and ERK phosphorylation, two pro-survival signaling pathways [122].

Recent studies have shown the potential application value of oleuropein in the treatment of lung cancer. Wang et al. showed that in H1299 lung cancer cells, oleuropein induced apoptosis via mitochondrial apoptotic cascade activated by p38 MAPK signaling [123]. More recently, it has been reported that oleuropein has the potential to inhibit cell motility in prostate cancer through the blocking of voltage-gated sodium channels (VGSC) due to the downregulation of mRNA expression in SCN9A, pore-forming α-subunits of the VGSC complex [124]. Furthermore, oleuropein’s inhibitory effect on cell motility was demonstrated in in vivo studies in mice (HR-1 mice) with UVB-induced skin damage and carcinogenesis. Orally administered oleuropein prevented a UVB-induced increase in skin thickness, and this effect was associated with the inhibition of MMP-13, MMP-2, MMP-9, and VEGF expression [125].

In addition, in triple-negative breast cancer cells [102] and osteosarcoma cells [126], treatment with oleuropein inhibited cell migration and invasion by activating autophagy. The anti-cancer effects of oleuropein have been also examined in pancreatic cancer cells. Gene expression analysis revealed that c-Jun and c-Fos were involved in oleuropein-induced apoptosis [127]. In esophageal cancer cells, oleuropein inhibited cell growth, in vitro and in vivo, in a xenograft tumor model through the inhibition of HIF-1α and the upregulation of BTG anti-proliferation factor 3 (BTG3) expression [128].

A low incidence of breast cancer in Mediterranean countries suggests that a high consumption of EVOO might confer this benefit. Mendez et al. have shown that oleuropein aglycone is the most potent EVOO phenolic compound involved in decreasing the viability of breast cancer cells [129]. The study was conducted on the human HER2-negative MCF-7 and HER2-positive SKBR3 BC cell lines. This last cell line, and MCF-7 cells transfected with HER2, were five times more sensitive to the effects of oleuropein aglycone than HER2-negative MCF-7 breast cancer cells, indicating a potential anti-tumor effect of oleuropein aglycone in HER2^+^ breast cancer. Interestingly, in a preclinical model, SKRB3 cells resistant to trastuzumab (SKBR3/Tzb100 cells) recovered their trastuzumab sensitivity when cultured in the presence of oleuropein aglycone. [130]. The overexpression of HER2 in breast cancer cells treated with trastuzumab was markedly suppressed by oleuropein aglycone exposure [130]. The same authors, in a subsequent paper, demonstrated that treatment with secoiridoid derivatives, such as ligstroside aglycone, deacetoxyoleuropein aglycone, and oleuropein glycoside in overexpressing HER2 breast cancer cells, was more significantly effective in its anti-cancer effect by suppressing fatty acid synthase (FASN), when compared to trastuzumab treatment [130]. The efficacy of treatment with oleuropein in combination with doxorubicin in a breast cancer tumor xenograft model has been also investigated [131]. Oleuropein/doxorubicin co-treatment in nude mice bearing MDA-MB-231-derived xenograft tumors induced a decrease in tumor volume and provoked apoptosis via the mitochondrial pathway [131]. Moreover, subsequent experimental studies have shown that oleuropein induced apoptosis in breast cancer cells by modulating NF-κB activation cascade [132], reducing PTP1B phosphatase activity [133], and modulating miR-21 and miR-155 expression [134].

In vitro and in vivo studies have shown that treatment of human neuroblastoma cells with oleuropein aglycone caused cell cycle arrest and autophagy through mTOR inhibition and AMPK activation [135]. The link between AMPK activation and mTOR inhibition was shown in an oleuropein-fed animal model, which showed decreased phospho-mTOR and increased phospho-AMPK levels, supporting the idea that autophagy activation by oleuropein aglycone proceeds through mTOR inhibition [135] (Table 3).

## 8. Ligstroside Aglycone

Ligstroside aglycone is the third of the four abundant and important phenols present in EVOO. To date, there have been few studies testing the biological activity of this compound. Ligstroside aglycone has been primarily used in several studies in which its possible anti-cancer effects have been investigated.

Although ligstroside aglycone has been shown to have moderate in vitro cytotoxicity against a panel of 39 human cancer cell lines [136], Mendez et al. reported that ligstroside aglycone induced apoptosis in breast cancer cells overexpressing HER2 [129]. In addition, Busnena et al. demonstrated that in the MDA-MB231 human breast cancer cell line, ligstroside aglycone treatment showed antimigratory activity through the inhibition of c-MET signaling, without any cytotoxicity to normal cells [137].

Finally, more recently, De Stefanis et al. have shown that exposure of HCC cells to a phenolic extract, essentially composed of a mixture of OC and ligstroside aglycone, induced cell death and autophagy and moreover, these antitumor effects could be enhanced by the addition of TNF-α [83] (Table 3).

## 9. Conclusions

Alongside its well-documented anti-inflammatory action linked to the inhibition of COX enzymes [14,108], preclinical studies have also defined the ability of secoiridoids to modulate the oxidative state of cells, carried out both as antioxidants and pro-oxidants in a dose-dependent manner [58,91,98,120]. The results obtained so far, in in vitro and in vivo cancer models, clearly show that secoiridoids can also exert anti-cancer activity due to their ability to induce ROS production.

Indeed, all compounds showed the ability to inhibit cell proliferation and induce apoptosis, although with different targets, depending on the tumor model. Secoiridoids inhibit essential pathways for proliferation, such as AKT and ERK [78,79,80,81,92,106,107,122], induce apoptosis by interfering with the expression of pro-apoptotic proteins, such as Bax [97], or anti-apoptotic Mcl1 and Bcl-xl proteins [79], modulate the autophagy pathway [83,103,104,126], and regulate metalloproteinases [79]. The action that they perform on the basic mechanisms of EMT, which results in tumor invasiveness and metastasis, is also relevant. In fact, while Tyr acts on the β-catenin and TGF-β pathways in breast cancer [104], in the same cancer model OC modulates the HER2/MET pathway and reduces vimentin by significantly inhibiting the recurrence rate [70], which is what makes these compounds exploitable in post-surgery therapy.

Of particular note are the studies conducted in combination with other chemotherapeutic drugs (i.e., OC plus lapatinib; OC plus Tamoxifen; Oleuropein plus cisplatin or trastuzumab; Tyr plus paclitaxel) that highlighted that the addition of secoiridoids resulted in synergistic effects in reducing tumor cell proliferation [66,67,100,120,130]. Furthermore, secoiridoids have not been found to be cytotoxic for healthy cells [58,81,106]. 

Many recent clinical trials, completed or in progress, have been developed for evaluating the effects of EVOO and the MD as a support for therapeutic protocols in different types of neoplasms. Studies are being conducted to evaluate improvements in metabolic function, body weight, and survival in prostate cancer patients (NCT03084913; NCT01083771), to study the prevention of breast cancer (NCT04174391; NCT02068092) and to test its ability to modulate side effects and alleviate cancer-related fatigue in patients undergoing chemotherapy (NCT03399331; NCT04534738).

A clinical study is currently underway to evaluate the effects of the dietary intake of olive oil rich in OC on disease progression in patients with chronic lymphocytic leukemia (CCL). One of purposes of this clinical trial is also to study the anti-cancer mechanism of the EVOO phenol OC in neoplasia (NCT04215367), which confirms the importance of the preclinical studies summarized and analyzed in this review.

The large number of significant results emerging from all the reported data make secoiridoids effective anti-cancer agents, both alone and in combination with other drugs, suitable for more significant use in future therapies.

## Figures and Tables

**Table 1:** Anti-cancer effects of Oleocanthal and Oleacein: in vitro and in vivo studies.

Oleocanthal				
Tumor	Model Cell Line	Molecular Target	Ref	In Vivo Model
**Colon Cancer**	SW480	↑ROS	[58]	
	HT29	↓COX2		
		↑γH2AX		
	HT29	↑AMP	[59]	HT29 cells CAM assay
	Caco	↓p-mTOR	[74]	
**Breast Cancer**	MCF-7	↓p-cMET	[61]	
	MDA-MB-231			
		↓AKT	[63]	MDA-MB-231 cells xenografts in athymic nude mice
		↓MAPK		
	BT-474	↓Brk/paxillin/Rac1		
	MCF-7	↑E-cadherin		
	MDA-MB-231	↑Zo-1		
		↓vimentin		
		↓ERα receptor		
	MCF-7	↓p-mTOR	[74]	
	T-47D			
	MDA-MB-231			
		↓c-MET	[66]	BT-474 orthotopic model in athymic nude mice
		↓HER2		
	BT-474	↓EGFR		
	SK-BR-3	↓AKT		
		↓PI3H		
		↓MAPK		
		↓STAT3		
		↓vimentin		
	MDA-MB-231	↑LMP	[75]	
	MCF-7	↓TRPC6	[72]	
	MDA-MB-231			
**Prostate Cancer**	PC3	↓p-cMET	[61]	
	PC3	↑LMP	[75]	
**HCC**	HepG2	↑ROS	[58]	
	Huh7	↓COX2		
	Hep3B	↑γH2AX		
	PLC/PRF/5			
	HepG2	↑AKT	[83]	
	Huh7	↑ERK		
	Hep3B	↑LC3-II/LC3-I		
		↓p62		
	HepG2	↓STA3	[84]	
	Huh7	↓JAK1, JAK2		
	HCCLM3	↓TWIST		
**Melanoma**		↓Mcl1	[80]	A375 cells xenografts in nude mice
		↓BCL-xl		
	A375	↓MMP9		
	A2058	↓MMP2		
		↓STAT3		
		↓JAK2		
**Lung Cancer**	A549	↓p-cMET	[71]	orthotopic model of A549 cells in athymic nude mice
	NCI-H322M	↓COX2		
**Pancreatic Cancer**	BxPC3	↑LMP	[75]	PNET RIP-Tag mice
**Multiple Myeloma**	ARH-77	↓AKT	[78]	
		↓ERK		
		↑p38		
		↓MIP-1α		
**Histiocytic Lymphoma**	U937	↓Hsp90	[82]	
		↓AKT		
		↓Cdk4		
		**Oleacein**		
**Tumor**	**Model Cell Line**	**Molecular Target**	**Ref**	**In Vivo Model**
**Non-melanoma Skin Cancer**	A43	↓AKT	[88]	
		↓ERK		
		↓B-Raf		
**Neuroblastoma**	SH-SY5Y	↑Bax	[89]	
		↑p53		
		↓Bcl2		
		↓STAT3		

**Table 2:** Anti-cancer effects of Hydroxytyrosol and Tyrosol: in vitro and in vivo studies.

Hydroxytyrosol				
Tumor	Model Cell Line	Molecular Target	Ref	In Vivo Model
**Prostate Cancer**		↓AKT	[106]	
		↓STA3		
	LNCaP	↓MCT4		
	C4-2	↑Bax		
		↑Bcl-2		
		↓NF-κB		
**Colon Cancer**	Caco-2	↓pERK	[92]	
		↓cyclin D1		
	DLD1	↑ROS	[93]	
	HT-29	↓EGFR	[94]	HT-29 cells xenografts in immunodeficient mice
	CaCo2	↑pCBL		
	Caco-2	↑CB1	[96]	
	LS180	↑CASP3	[97]	
		↑Bax		
**HCC**	HepG2	↓AKT	[107]	orthotopic HCC model in 4–6-week old nude mice
	Huh7	↓NF-κB		
	Hep3B			
	SK-HEP-1			
	HepG2	↓FAS	[108]	
	Hep3B	↓FPPS		
**Breast Cancer**	MCF-7	↓PGC1a/Nrf2	[98]	
		↓PGC1a/ERRa		
	MCF-7	↑ROS	[100]	
	MDA-MB-231	↓DNA damage		
	MCF-7	↑LC3-II/LC3-I	[104]	
	MDA-MB-231	↓p62		
	T47D			
	SUM159PT	↓β-catenin	[105]	breast tumor-bearing rats
	MDA-MB-231	↓cyclin D1		
	Hs578T	↓Snail		
	BT549	↓vimentin		
		↓SMAD2/3		
**Tyrosol**				
**Tumor**	**Model Cell Line**	**Molecular target**	**Ref**	**In Vivo Model**
**Glioblastoma**	U-87 MG	↓pJNK	[109]	
		↓pERK		
		↓NF-κB		
		↓COX2		

**Table 3:** Anti-cancer effects of Oleuropein and Ligstroside: in vitro and in vivo studies.

Oleuropein				
Tumor	Model Cell Line	Molecular Target	Ref	In Vivo Model
**Melanoma**	A375	↓GLUT1	[112]	
		↓PKM2		
		↓MCT4		
**Colon Cancer**	SW620	↓FAS	[113]	
	HT-29			
	HT-29	↓HIF-1α	[116]	
		↑p53		
		↑PPARγ		
		↓NF-κB		
	HT-29	↓DNA damage	[117]	Azoxymethane (AOM)-treated mice
	HT-29	↓IL-6		AOM and d extran sulfate sodium (DSS)-treated mice
		↓IFN-γ		
		↓TNF-α		
		↓IL-17	[118]	
		↓COX-2		
		↓NF-κB		
		↓Wnt/β-catenin		
		↑STAT3		
**Chronic Myeloid Leukemia**	HL60	↑Apoptosis	[115]	

**HCC**	HepG2	↑Bax	[119]	
		↑Bcl-2		
		↓AKT		
	HepG2	↑MMP-7	[120]	
**Neuroblastoma**	SH-SY5Y	↓cylin D1	[121]	
		↓cylin D2		
		↓cyclin D3		
		↓CDK4		
		↓CDK6		
		↑p53		
		↑CDKN2A		
		↑CDKN2B		
		↑CDKN1A		
**Thyroid Cancer**	TPC-1	↓p-AKT	[122]	
	BCPAP	↓p-ERK		
		↑ROS		
**Lung Cancer**	H1299	↑p38	[123]	
**Prostate Cancer**	MAT-LyLu	↓SCN9A	[124]	
**Skin Cancer**		↓VEGF	[125]	skin damage and carcinogenesis in hairless mice exposed to UVB irradiation
		↓MMP-2		
		↓MMP-9		
		↓MMP-13		
		↓COX-2		
**Breast Cancer**	MDA-MB-231	↑LC3II	[103]	
		↓p62		
	MCF-7	↓HER2	[113]	
	SKBR3	↓cyclin D1	[131]	MDA-MB-231 cells xenografts in nude mice
		↓NF-κB		
		↓Bcl-2		
		↓survivin		
	MCF-7	↓NF-κB	[132]	
	MDA-MB-231			
	MCF-7	↓PTP1B	[133]	
	MCF-7	↓miR-21	[134]	
		↓miR-155		
** Osteosarcoma **	143B OS	↑LC3-II/LC3-I	[126]	
**Pancreatic Cancer**	MIAPaCa-2	↑c-Jun	[127]	
	BxPC-3	↑c-Fos		
	CFPAC-1			
**Esophageal Cancer**	EC	↓HIF-1α	[128]	EC cells xenografts in nude mice
		↑BTG3		
**Ligstroside**				
**Tumor**	**Model Cell Line**	**Molecular Target**	**Ref**	**In Vivo Model**
**Breast Cancer**	MDA-MB231	↓c-MET	[137]	
**HCC**		↑AKT	[83]	
		↑ERK		
	HepG2	↑mTOR		
	Huh7	↑p70		
	Hep3B	↑P-4E-BP1		
		↑LC3-II/LC3-I		
		↑Beclin-1		
		↑p62		

## References

[1] Bray F., Ferlay J., Soerjomataram I., Siegel R.L., Torre L.A., Jemal A. (2018). Global cancer statistics 2018: GLOBOCAN estimates of incidence and mortality worldwide for 36 cancers in 185 countries. CA Cancer J. Clin..

[2] Danaei G., Ding E.L., Mozaffarian D., Taylor B., Rehm J., Murray C.J.L., Ezzati M. (2009). The preventable causes of death in the United States: Comparative risk assessment of dietary; lifestyle; and metabolic risk factors. PLoS Med..

[3] Donaldson M. (2004). Nutrition and cancer: A review of the evidence for an anti-cancer diet. Nutr. J..

[4] Trichopoulou A., Critselis E. (2004). Mediterranean diet and longevity. Eur. J. Cancer Prev..

[5] Bach-Faig A., Berry E.M., Lairon D., Reguant J., Trichopoulou A., Dernini S., Medina F.X., Battino M., Belahsen R., Miranda G. (2011). Mediterranean Diet Foundation Expert Group; Mediterranean diet pyramid today. Sci. Cult. Updates Public Health Nutr..

[6] Mentella M.C., Scaldaferri F., Ricci C., Gasbarrini A., Miggiano G.A.D. (2019). Cancer and Mediterranean Diet: A review. Nutrients.

[7] Grosso G., Buscemi S., Galvano F., Mistretta A., Marventano S., La Vela V., Drago F., Gangi S., Basile F., Biondi A. (2013). Med-iterranean diet and cancer: Epidemiological evidence and mechanism of selected aspects. BMC Surg..

[8] Schwingshack L., Hoffmann G. (2015). Adherence to Mediterranean diet and risk of cancer: An updated systematic review and meta-analysis of observational studies. Cancer Med..

[9] Owen R., Giacosa A., Hull W., Haubner R., Spiegelhalder B., Bartsch H. (2000). The antioxidant/anticancer potential of phenolic compounds isolated from olive oil. Eur. J. Cancer.

[10] Servili M., Esposto S., Fabiani R., Urbani S., Taticchi A., Mariucci F., Selvaggini R., Montedoro G.F. (2009). Phenolic compounds in olive oil: Antioxidant, health and organoleptic activities according to their chemical structure. Inflammopharmacology.

[11] Bulotta S., Celano M., Lepore S.M., Montalcini T., Pujia A., Russo D. (2014). Beneficial effects of the olive oil phenolic components oleuropein and hydroxytyrosol: Focus on pro-tection against cardiovascular and metabolic diseases. J. Transl. Med..

[12] Cárdeno A., Sánchez-Hidalgo M., Alarcón-De-La-Lastra C. (2013). An up-date of olive oil phenols in inflammation and cancer: Molecular mechanisms and clinical implications. Curr. Med. Chem..

[13] Rigacci S., Stefani M. (2016). Nutraceutical Properties of Olive Oil Polyphenols. An Itinerary from Cultured Cells through Animal Models to Humans. Int. J. Mol. Sci..

[14] Beauchamp G.K., Keast R.S.J., Morel D., Lin J., Pika J., Han Q., Lee C.-H., Smith A.B., Breslin P.A.S. (2005). Phytochemistry: Ibuprofen-like activity in extra-virgin olive oil. Nature.

[15] Iacono A., Gómez R., Sperry J., Conde J., Bianco G., Meli R., Gómez-Reino J.J., Smith A.B., Gualillo O. (2010). Effect of oleocanthal and its derivatives on inflammatory response induced by lipopolysaccharide in a murine chondrocyte cell line. Arthritis Rheum..

[16] Li W., Sperry J.B., Crowe A., Trojanowski J.Q., Iii A.B.S., Lee V.M.-Y. (2009). Inhibition of tau fibrillization by oleocanthal via reaction with the amino groups of tau. J. Neurochem..

[17] Pitt J., Roth W., Lacor P., Smith A.B., Blankenship M., Velasco P., De Felice F., Breslin P., Klein W.L. (2009). Alzhei-mer’s-associated Aβ oligomers show altered structure; immunoreactivity and synaptotoxicity with low doses of oleocanthal. Toxicol. Appl. Pharmacol..

[18] Qosa H., Batarseh Y.S., Mohyeldin M.M., El Sayed K.A., Keller J.N., Kaddoumi A. (2015). Oleocanthal Enhances Amyloid-β Clearance from the Brains of TgSwDI Mice and in Vitro across a Human Blood-Brain Barrier Model. ACS Chem. Neurosci..

[19] Grilo F., Sedaghat S., Di Stefano V., Schicchi R., Caruso T., Lo Bianco R. (2021). Tree Planting Density and Canopy Position Affect ‘Cerasuola’ and ‘Koroneiki’ Olive Oil Quality. Horticulturae.

[20] Huang Y.L., Oppong M.B., Guo Y., Wang L.Z., Fang S.M., Deng Y.R., Gao X.M. (2019). The Oleaceae family: A source of secoiridoids with multiple biological activities. Fitoterapia.

[21] Boussahel S., Di Stefano V., Muscarà C., Cristani M., Melilli M.G. (2020). Phenolic Compounds Characterization and Antioxidant Properties of Monocultivar Olive Oils from Northeast Algeria. Agriculture.

[22] Grilo F., Novara M.E., D’Oca M.C., Rubino S., Bianco R.L., Di Stefano V. (2019). Quality evaluation of extra-virgin olive oils from Sicilian genotypes grown in a high-density system. Int. J. Food Sci. Nutr..

[23] Perez J.A., Hernández J.M., Trujillo J.M., Lopez H. (2005). Iridoids and secoiridoids from Oleaceae. Bioact. Nat. Prod..

[24] Di Stefano V., Melilli M.G. (2020). Effect of storage on quality parameters and phenolic content of Italian extra-virgin olive oils. Nat. Prod. Res..

[25] Diamantakos P., Velkou A., Killday K.B., Gimisis T., Melliou E., Magiatis P. (2015). Oleokoronal and oleomissional: New major phenolic ingredients of extra virgin olive oil. Olivae.

[26] Angelis A., Antoniadi L., Stathopoulos P., Halabalaki M., Skaltsounis L.A. (2018). Oleocanthalic and Oleaceinic acids: New compounds from Extra Virgin Olive Oil (EVOO). Phytochem. Lett..

[27] Montedoro G., Servili M., Baldioli M., Selvaggini R., Miniati E., Macchioni A. (1993). Simple and hydrolyzable compounds in virgin olive oil. 3. Spectroscopic characterizations of the secoiridoid derivatives. J. Agric. Food Chem..

[28] Andrewes P., Busch J., de Joode T., Groenewegen A., Alexandre H.J. (2003). Sensory properties of virgin olive oil polyphenols: Identification of deacetoxy-ligstroside aglycon as a key contributor to pungency Agric. Food Chem..

[29] Impellizzeri J., Lin J. (2006). A Simple High-Performance Liquid Chromatography Method for the Determination of Throat-Burning Oleocanthal with Probated Antiinflammatory Activity in Extra Virgin Olive Oils. J. Agric. Food Chem..

[30] Romani A., Pinelli P., Mulinacci N., Galardi C., Vincieri F.F., Liberatore L., Cichelli A. (2001). HPLC and HRGC Analyses of Pol-yphenols and Secoiridoid in Olive Oil. Chromatographia.

[31] Adhami H.-R., Zehl M., Dangl C., Dorfmeister D., Stadler M., Urban E., Hewitson P., Ignatova S., Krenn L. (2015). Preparative isolation of oleocanthal, tyrosol, and hydroxytyrosol from olive oil by HPCCC. Food Chem..

[32] Angelis A., Michailidis D., Antoniadi L., Stathopoulos P., Tsantila V., Nuzillard J.M., Renault J.H., Skaltsounis L.A. (2021). Pilot continuous centrifugal liquid-liquid extraction of extra virgin olive oil biophenols and gram-scale recovery of pure oleocanthal, oleacein, MFOA, MFLA and hydroxytyrosol. Sep. Purif. Technol..

[33] Smith A.B., Han Q., Breslin P.A.S., Beauchamp G.K. (2005). Synthesis and Assignment of Absolute Configuration of (−)-Oleocathal: A Potent, Naturally Occurring Non-steroidal Anti-inflammatory and Anti-oxidant Agent Derived from Extra Virgin Olive Oils. Org. Lett..

[34] English B.J., Williams R.M. (2009). Synthesis of (±)-oleocanthal via a tandem intramolecular Michael cyclization–HWE olefination. Tetrahedron Lett..

[35] Kuch J.T.B., O’Connor P.D., Hügel H., Brimble M.A. (2009). Synthetic studies towards the anti-inflammatory agent, oleocanthal using a Johnson–Claisen (orthoester) rearrangement strategy. ARKIVOC.

[36] Takahashi K., Morita H., Honda T. (2012). Formal synthesis of (−)-oleocanthal by means of a SmI2-promoted intramolecular cou-pling of bromoalkyne with α,β-unsaturated ester. Tetrahedron Lett..

[37] Valli M., Peviani E.G., Porta A., D’Alfonso A., Zanoni G., Vidari G. (2013). A Concise and Efficient Total Synthesis of Oleocan-thal. Eur. J. Org. Chem..

[38] Sarikaki G., Christoforidou N., Gaboriaud-Kolar N., Smith A.B., Kostakis I.K., Skaltsounis A.L. (2020). Biomimetic Synthesis of Oleocanthal, Oleacein, and Their Analogues Starting from Oleuropein, A Major Compound of Olive Leaves. J. Nat. Prod..

[39] Calero J., Martínez L., García-Granados A. (1994). Procedimiento Deaprovechamiento del Alpechin Para la Obtención Deácidos; Fenoles; Alcohols y Derivados Mediante Extracciónen Contracorriente.

[40] Cuomo J., Rabovskiy A.B. (2001). Antioxidant Compositions Extracted from Olives and Olive by-Products. U.S. Patent.

[41] Crea R. (2002). Method of Obtaining a Hydroxytyrosol-Rich Composition from Vegetation Water. U.S. Patent.

[42] Brenes M., Castro A. (2003). Procedure is for Obtaining Phenolics Extract with High Concentration of Anti-Oxidants and Involves Ul-tra-Filtration of Solutions Derived from Preparation Process of Preserved Table Olives.

[43] Fernández-Bolaños J., Rodríguez G., Rodríguez R., Guillén R., Jimenez-Araujo A. (2006). Extraction of interesting organic compounds from olive oil waste. Grasas Aceites.

[44] Yasemi M., Heydarinasab A., Rahimi M., Ardjmand M. (2017). Microchannels Effective Method for the Extraction of Oleuropein Compared with Conventional Methods. J. Chem..

[45] Sengling Cebin Coppa C.F., Gonçalves B.L., Hwa S., Lee I., Martinelli V., Nunes R., Gonçalves C.B., Costa Rodrigues C.E., Oliveira C.A.F. (2020). Extraction of oleuropein from olive leaves and applicability in foods. Qual. Assur. Saf. Crop. Foods.

[46] Rosa G.S., Vanga S.K., Gariepy Y., Raghavan V. (2019). Comparison of microwave; ultrasonic and conventional techniques for ex-traction of bioactive compounds from olive leaves *Olea europaea* L.. Innov. Food Sci. Emerg. Technol..

[47] Khemakhem I., Gargouri O.D., Dhouib A., Ayadi M.A., Bouaziz M. (2017). Oleuropein rich extract from olive leaves by combining microfiltration, ultrafiltration and nanofiltration. Sep. Purif. Technol..

[48] Zhang Q.W., Lin L.G., Ye W.C. (2018). Techniques for extraction and isolation of natural products: A comprehensive review. Chin. Med..

[49] Baldino L., Della Porta G., Osseo L.S., Reverchon E., Adami R. (2018). Concentrated oleuropein powder from olive leaves using alcoholic extraction and supercritical CO2 assisted extraction. J. Supercrit. Fluids.

[50] Lamprou G.K., Vlysidis A., Tzathas K., Vlyssides A.G. (2020). Statistical optimization and kinetic analysis of the extraction of phenolic compounds from olive leaves. J. Chem. Technol. Biotechnol..

[51] Murowaniecki D.O., Lorini A., Antunes B.d.F., Oliveira R.M., Zambiazi R. (2020). Oleuropein: Methods for extraction; purifying and applying. Rev. Ceres..

[52] Cicerale S., Breslin P.A., Beauchamp G.K., Keast R. (2009). Sensory Characterization of the Irritant Properties of Oleocanthal, a Natural Anti-Inflammatory Agent in Extra Virgin Olive Oils. Chem. Senses.

[53] Abuznait A.H., Qosa H., Busnena B.A., El Sayed K.A., Kaddoumi A. (2013). Olive-Oil-Derived Oleocanthal Enhances β-Amyloid Clearance as a Potential Neuroprotective Mechanism against Alzheimer’s Disease: In Vitro and in Vivo Studies. ACS Chem. Neurosci..

[54] Gachons C., Uchida K., Bryant B., Shima A., Sperry J.B., Dankulich-Nagrudny L., Tominaga M., Smith A.B. (2011). Unusual pungency from extra-virgin olive oil is attributable to restricted spatial expression of the receptor of oleocanthal. Neuroscience.

[55] García-Villalba R., Carrasco-Pancorbo A., Nevedomskaya E., Mayboroda O.A., Deelder A.M., Segura-Carretero A., Fernández-Gutiérrez A. (2010). Exploratory analysis of human urine by LC–ESI-TOF MS after high intake of olive oil: Understanding the metabolism of polyphenols. Anal. Bioanal. Chem..

[56] Romero C., Medina E., Vargas J., Brenes M., De Castro A. (2007). In Vitro Activity of Olive Oil Polyphenols against Helicobacter pylori. J. Agric. Food Chem..

[57] http://www.chemspider.com/Chemical-Structure.9827154.html.

[58] Cusimano A., Balasus D., Azzolina A., Augello G., Emma M.R., Di Sano C., Gramignoli R., Strom S.C., McCubrey J.A., Montalto G. (2017). Oleocanthal exerts antitumor effects on human liver and colon cancer cells through ROS genera-tion. Int. J. Oncol..

[59] Khanal P., Oh W.-K., Yun H.J., Namgoong G.M., Ahn S.-G., Kwon S.-M., Choi H.S. (2011). p-HPEA-EDA, a phenolic compound of virgin olive oil, activates AMP-activated protein kinase to inhibit carcinogenesis. Carcinogenesis.

[60] Hardie D.G., Iwadate Y., Yumura S. (2004). The AMP-activated protein kinase pathway-new players upstream and downstream. J. Cell Sci..

[61] Elnagar A.Y., Sylvester P.W., El Sayed K.A. (2011). (−)-Oleocanthal as a c-Met Inhibitor for the Control of Metastatic Breast and Prostate Cancers. Planta Med..

[62] Ho-Yen C.M., Jones J.L., Kermorgant S. (2015). The clinical and functional significance of c-Met in breast cancer: A review. Breast Cancer Res..

[63] Akl M.R., Ayoub N.M., Mohyeldin M.M., Busnena B.A., Foudah A.I., Liu Y.Y., Ei Sayed K.A. (2014). Olive phenolics as c-Met inhibi-tors: (−)-Oleocanthal attenuates cell proliferation; invasiveness; and tumor growth in breast cancer models. PLoS ONE.

[64] Mittal V. (2018). Epithelial Mesenchymal Transition in Tumor Metastasis. Annu. Rev. Pathol. Mech. Dis..

[65] Mohyeldin M.M., Akl M.R., Mady M.S., Dragoi A.M., Dykes S., Cardelli J.A., El Sayed K.A. (2016). The oleocanthal-based homovanillyl sinapate as a novel c-Met inhibitor. Oncotarget.

[66] Ayoub N.M., Siddique A.B., Ebrahim H.Y., Mohyeldin M.M., El Sayed K.A. (2017). The olive oil phenolic (−)-oleocanthal modu-lates estrogen receptor expression in luminal breast cancer in vitro and in vivo and synergizes with tamoxifen treatment. Eur. J. Pharmacol..

[67] Siddique A.B., Ebrahim H.Y., Akl M.R., Ayoub N.M., Goda A.A., Mohyeldin M.M., Nagumalli S.K., Hananeh W.M., Liu Y.Y., Meyer S.A. (2019). (−)-Oleocanthal as a Dual c-MET-COX2 Inhibitor for the Control of Lung Cancer. Nutrients.

[68] Samiee S., Berardi P., Bouganim N., Vandermeer L., Arnaout A., Dent S., Mirsky D., Chasen M., Caudrelier J.M., Clemons M. (2012). Excision of the primary tumor in patients with metastatic breast cancer: A clinical dilemma. Curr. Oncol..

[69] Isakoff S.J. (2010). Triple negative breast cancer: Role of specific chemotherapy agents. Cancer J..

[70] Siddique A.B., Ayoub N.M., Tajmim A., Meyer S.A., Hill R.A., El Sayed K.A. (2019). (−)-Oleocanthal Prevents Breast Cancer Lo-coregional Recurrence After Primary Tumor Surgical Excision and Neoadjuvant Targeted Therapy in Orthotopic Nude Mouse Models. Cancers.

[71] Siddique A.B., Kilgore P., Tajmim A., Singh S.S., Meyer S.A., Jois S., Cvek U., Trutschl M., El Sayed K.A. (2020). (−)-Oleocanthal as a Dual c-MET-COX2 Inhibitor for the Control of Lung Cancer. Nutrients.

[72] Diez-Bello R., Jardin I., Lopez J., El Haouari M., Ortega-Vidal J., Altarejos J., Salido G., Salido S., Rosado J.A. (2019). (−)-Oleocanthal inhibits proliferation and migration by modulating Ca^2+^ entry through TRPC6 in breast cancer cells. Biochim. Biophys. Acta Bioenergy.

[73] Miller T.W., Rexer B.N., Garrett J.T., Arteaga C.L. (2011). Mutations in the phosphatidylinositol 3-kinase pathway: Role in tumor progression and therapeutic implications in breast cancer. Breast Cancer Res..

[74] Khanfar M.A., Bardaweel S.K., Akl M.R., El Sayed K.A. (2015). Olive Oil-derived Oleocanthal as Potent Inhibitor of Mammalian Target of Rapamycin: Biological Evaluation and Molecular Modeling Studies. Phytother. Res..

[75] Legendre O., Breslin P.A., Foster D.A. (2015). (−)-Oleocanthal rapidly and selectively induces cancer cell death via lysosomal membrane permeabilization. Mol. Cell. Oncol..

[76] Wang F., Gómez-Sintes R., Boya P. (2018). Lysosomal membrane permeabilization and cell death. Traffic.

[77] Goren L., Zhang G., Kaushik S., Breslin P.A.S., Du Y.N., Foster D.A. (2019). (−)-Oleocanthal and (−)-oleocanthal-rich olive oils in-duce lysosomal membrane permeabilization in cancer cells. PLoS ONE.

[78] Scotece M., Gómez R., Conde J., Lopez V., Gómez-Reino J.J., Lago F., Smith A.B., Gualillo O. (2013). Oleocanthal inhibits proliferation and MIP-1α expression in human multiple myeloma cells. Curr. Med. Chem..

[79] Fogli S., Arena C., Carpi S., Polini B., Bertini S., Digiacomo M., Gado F., Saba A., Saccomanni G., Breschi M.C. (2016). Cytotoxic Activity of Oleocanthal Isolated from Virgin Olive Oil on Human Melanoma Cells. Nutr. Cancer.

[80] Lopez-Bergami P., Fitchman B., Ronai Z.A. (2007). Understanding Signaling Cascades in Melanoma. Photochem. Photobiol..

[81] Gu Y., Wang J., Peng L. (2016). (−)-Oleocanthal exerts anti-melanoma activities and inhibits STAT3 signaling pathway. Oncol. Rep..

[82] Margarucci L., Cassiano C., Mozzicafreddo M., Angeletti M., Riccio R., Monti M.C., Tosco A., Casapullo A. (2013). Chemical proteomics-driven discovery of oleocanthal as an Hsp90 inhibitor. Chem. Commun..

[83] De Stefanis D., Scimè S., Accomazzo S., Catti A., Occhipinti A., Bertea C.M., Costelli P. (2019). Anti-Proliferative Effects of an Extra-Virgin Olive Oil Extract Enriched in Ligstroside Aglycone and Oleocanthal on Human Liver Cancer Cell Lines. Cancers.

[84] Pei T., Meng Q., Han J., Sun H., Li L., Song R., Sun B., Pan S., Liang D., Liu L. (2016). (−)-Oleocanthal inhibits growth and me-tastasis by blocking activation of STAT3 in human hepatocellular carcinoma. Oncotarget.

[85] Ünsal Ü.Ü., Mete M., Aydemir I., Duransoy Y.K., Umur A.Ş., Tuglu M.I. (2020). Inhibiting effect of oleocanthal on neuroblastoma cancer cell proliferation in culture. Biotech. Histochem..

[86] Briante R., Patumi M., Terenziani S., Bismuto E., Febbraio F., Nucci R.J. (2002). Olea europaea L. Leaf Extract and Derivatives: Antioxidant PropertiesAgric. Food Chem..

[87] Czerwinska M., Kiss A.K., Naruszewicz M. (2012). A comparison ofantioxidant activities of oleuropein and its dialdehydic deriva-tive from olive oil, oleacein. Food Chem..

[88] Polini B., Digiacomo M., Carpi S., Bertini S., Gado F., Saccomanni G., Macchia M., Nieri P., Manera C., Fogli S. (2018). Oleocanthal and oleacein contribute to the in vitro therapeutic potential of extra virgin oil-derived extracts in non-melanoma skin cancer. Toxicol. In Vitro.

[89] Cirmi S., Celano M., Lombardo G.E., Maggisano V., Procopio A., Russo D., Navarra M. (2020). Oleacein inhibits STAT3; acti-vates the apoptotic machinery; and exerts anti-metastatic effects in the SH-SY5Y human neuroblastoma cells. Food Funct..

[90] Karković Marković A., Torić J., Barbarić M., Jakobušić Brala C. (2019). Hydroxytyrosol, Tyrosol and Derivatives and Their Poten-tial Effects on Human Health. Molecules.

[91] Fabiani R., Sepporta M.V., Rosignoli P., De Bartolomeo A., Crescimanno M., Morozzi G. (2012). Anti-proliferative and pro-apoptotic activities of hydroxytyrosol on different tumour cells: The role of extracellular production of hydrogen peroxide. Eur. J. Nutr..

[92] Corona G., Deiana M., Incani A., Vauzour D., Dessi M.A., Spencer J.P.E. (2009). Hydroxytyrosol inhibits the proliferation of hu-man colon adenocarcinoma cells through inhibition of ERK1/2 and cyclin D1. Mol. Nutr. Food Res..

[93] Sun L., Luo C., Liu J. (2014). Hydroxytyrosol induces apoptosis in human colon cancer cells through ROS generation. Food Funct..

[94] Terzuoli E., Giachetti A., Ziche M., Donnini S. (2016). Hydroxytyrosol, a product from olive oil, reduces colon cancer growth by enhancing epidermal growth factor receptor degradation. Mol. Nutr. Food Res..

[95] Wang D., Wang H., Ning W., Backlund M.G., Dey S.K., Dubois R.N. (2008). Loss of Cannabinoid Receptor 1 Accelerates Intestinal Tumor Growth. Cancer Res..

[96] Di Francesco A., Falconi A., Di Germanio C., Di Bonaventura M.V.M., Costa A., Caramuta S., Del Carlo M., Compagnone D., Dainese E., Cifani C. (2015). Extravirgin olive oil up-regulates CB1 tumor suppressor gene in human colon cancer cells and in rat colon via epigenetic mechanisms. J. Nutr. Biochem..

[97] Hormozi M., Marzijerani A.S., Baharvand P. (2020). Effects of Hydroxytyrosol on Expression of Apoptotic Genes and Activity of Antioxidant Enzymes in LS180 Cells. Cancer Manag. Res..

[98] Calahorra J., Martínez-Lara E., De Dios C., Siles E. (2018). Hypoxia modulates the antioxidant effect of hydroxytyrosol in MCF-7 breast cancer cells. PLoS ONE.

[99] Calahorra J., Martínez-Lara E., Granadino-Roldán J.M., Martí J.M., Cañuelo A., Blanco S., Oliver F.J., Siles E. (2020). Crosstalk between hydroxytyrosol, a major olive oil phenol, and HIF-1 in MCF-7 breast cancer cells. Sci. Rep..

[100] El-Azem N., Pulido-Moran M., Ramirez-Tortosa C.L., Quiles J.L., Cara F.E., Sanchez-Rovira P., Granados-Principal S., Ramirez-Tortosa M. (2019). Modulation by hydroxytyrosol of oxidative stress and antitumor activities of paclitaxel in breast cancer. Eur. J. Nutr..

[101] Osman M.A., Elkady M. (2017). A Prospective Study to Evaluate the Effect of Paclitaxel on Cardiac Ejection Fraction. Breast Care.

[102] Schlitt A., Jordan K., Vordermark D., Schwamborn J., Langer T., Thomssen C. (2014). Cardiotoxicity and Oncological Treatments. Dtsch. Aerzteblatt Online.

[103] Lu H.-Y., Zhu J.-S., Zhang Z., Shen W.-J., Jiang S., Long Y.-F., Wu B., Ding T., Huan F., Wang S.-L. (2020). Hydroxytyrosol and Oleuropein Inhibit Migration and Invasion of MDA-MB-231 Triple-Negative Breast Cancer Cell via Induction of Autophagy. Anti-Cancer Agents Med. Chem..

[104] Lu H.Y., Zhu J.S., Xie J., Zhang Z., Zhu J., Jiang S., Shen W.J., Wu B., Ding T., Wang S.L. (2020). Hydroxytyrosol and Oleuropein Inhibit Migration and Invasion via Induction of Autophagy in ER-Positive Breast Cancer Cell Lines (MCF-7 and T47D). Nutr. Cancer.

[105] Cruz-Lozano M., González-González A., Marchal J.A., Muñoz-Muela E., Molina M.P., Cara F.E., Brown A.M., García-Rivas G., Hernández-Brenes C., Lorente J.A. (2019). Hydroxytyrosol inhibits cancer stem cells and the metastatic capacity of triple-negative breast cancer cell lines by the simultaneous targeting of epithelial-to-mesenchymal transition, Wnt/β-catenin and TGFβ signaling pathways. Eur. J. Nutr..

[106] Zubair H., Bhardwaj A., Ahmad A., Srivastava S.K., Khan M.A., Patel G.K., Singh S., Singh A.P. (2017). Hydroxytyrosol Induces Apoptosis and Cell Cycle Arrest and Suppresses Multiple Oncogenic Signaling Pathways in Prostate Cancer Cells. Nutr. Cancer.

[107] Zhao B., Ma Y., Xu Z., Wang J., Wang F., Wang D., Pan S., Wu Y., Pan H., Xu D. (2014). Hydroxytyrosol, a natural molecule from olive oil, suppresses the growth of human hepatocellular carcinoma cells via inactivating AKT and nuclear factor-kappa B pathways. Cancer Lett..

[108] Tutino V., Caruso M.G., Messa C., Perri E., Notarnicola M. (2012). Antiproliferative, antioxidant and anti-inflammatory effects of hydroxytyrosol on human hepatoma HepG2 and Hep3B cell lines. Anticancer Res..

[109] Lamy S., Ben Saad A., Zgheib A., Annabi B. (2016). Olive oil compounds inhibit the paracrine regulation of TNF-α-induced endo-thelial cell migration through reduced glioblastoma cell cyclooxygenase-2 expression. J. Nutr. Biochem..

[110] Shamshoum H., Vlavcheski F., Tsiani E. (2017). Anticancer effects of oleuropein. BioFactors.

[111] Torić J., Marković A.K., Brala C.J., Barbarić M. (2019). Anticancer effects of olive oil polyphenols and their combinations with an-ticancer drugs. Acta Pharm..

[112] Ruzzolini J., Peppicelli S., Bianchini F., Andreucci E., Urciuoli S., Romani A., Tortora K., Caderni G., Nediani C., Calorini L. (2020). Cancer Glycolytic Dependence as a New Target of Olive Leaf Extract. Cancers.

[113] Notarnicola M., Pisanti S., Tutino V., Bocale D., Rotelli M.T., Gentile A., Memeo V., Bifulco M., Perri E., Caruso M.G. (2010). Effects of olive oil polyphenols on fatty acid synthase gene expression and activity in human colorectal cancer cells. Genes Nutr..

[114] Menendez J.A., Vazquez-Martin A., Colomer R., Brunet J., Carrasco-Pancorbo A., Garcia-Villalba R., Fernan-dez-Gutierrez A., Segura-Carretero A. (2007). Olive oil’s bitter principle reverses acquired autoresistance to trastuzumab (Her-ceptin) in HER2-overexpressing breast cancer cells. BMC Cancer.

[115] Anter J., Fernández-Bedmar Z., Villatoro-Pulido M., Demyda-Peyras S., Moreno-Millán M., Alonso-Moraga A., Muñoz-Serrano A., Luque de Castro M.D. (2011). A pilot study on the DNA-protective, cytotoxic, and apoptosis-inducing properties of olive-leaf extracts. Mutat Res..

[116] Cárdeno A., Sánchez-Hidalgo M., Rosillo M.A., Alarcón-De-La-Lastra C. (2013). Oleuropein, a Secoiridoid Derived from Olive Tree, Inhibits the Proliferation of Human Colorectal Cancer Cell Through Downregulation of HIF-1α. Nutr. Cancer.

[117] Sepporta M.V., Fuccelli R., Rosignoli P., Ricci G., Servili M. (2016). Oleuropein prevents azoxymethane-induced colon crypt dys-plasia and leukocytes DNA damage in A/J mice. J. Med. Food.

[118] Giner E., Recio M.C., Ríos J.L., Cerdá-Nicolás J.M., Giner R.M. (2015). Chemopreventive effect of oleuropein in colitis-associated colorectal cancer in c57bl/6 mice. Mol. Nutr. Food Res..

[119] Yan C.-M., Chai E.-Q., Cai H.-Y., Miao G.-Y., Ma W. (2015). Oleuropein induces apoptosis via activation of caspases and suppression of phosphatidylinositol 3-kinase/protein kinase B pathway in HepG2 human hepatoma cell line. Mol. Med. Rep..

[120] Sherif I.O., Al-Gayyar M.M. (2018). Oleuropein potentiates anti-tumor activity of cisplatin against HepG2 through affecting proNGF/NGF balance. Life Sci..

[121] Secme M., Eroglu C., Dodurga Y., Bagc G. (2016). Investigation of anticancer mechanism of oleuropein via cell cycle and apoptotic pathways in SH-SY5Y neuroblastoma cells. Gene.

[122] Bulotta S., Corradino R., Celano M., Maiuolo J., D’Agostino M., Oliverio M., Procopio A., Filetti S., Russo D. (2013). Antioxidant and antigrowth action of peracetylated oleuropein in thyroid cancer cells. J. Mol. Endocrinol..

[123] Wang W., Wu J., Zhang Q., Li X., Zhu X., Wang Q., Cao S., Du L. (2019). Mitochondria-mediated apoptosis was induced by oleuropein in H1299 cells involving activation of p38 MAP kinase. J. Cell. Biochem..

[124] Aktas H.G., Ayan H. (2020). Oleuropein: A Potential Inhibitor for Prostate Cancer Cell Motility by Blocking Voltage-Gated Sodium Channels. Nutr. Cancer.

[125] Kimura Y., Sumiyoshi M. (2009). Olive leaf extract and its main component oleuropein prevent chronic ultraviolet B radiation-induced skin damage and carcinogenesis in hairless mice. J. Nutr..

[126] Przychodzen P., Wyszkowska R., Gorzynik-Debicka M., Kostrzewa T., Kuban-Jankowska A., Gorska-Ponikowska M. (2019). An-ticancer Potential of Oleuropein, the Polyphenol of Olive Oil, with 2-Methoxyestradiol, Separately or in Combination, in Human Osteosarcoma Cells. Anticancer Res..

[127] Goldsmith C.D., Bond D.R., Jankowski H., Weidenhofer J., Stathopoulos C.E., Roach P.D., Scarlett C.J. (2018). The Olive Bio-phenols Oleuropein and Hydroxytyrosol Selectively Reduce Proliferation, Influence the Cell Cycle and Induce Apoptosis in Pancreatic Cancer Cells. Int. J. Mol. Sci..

[128] Fengli Z., Mei Z. (2019). Oleuropein inhibits esophageal cancer through hypoxic suppression of BTG3 mRNA. Food Funct..

[129] Menendez J.A., Vazquez-Martin A., Garcia-Villalba R., Carrasco-Pancorbo A., Oliveras-Ferraros C., Fernandez-Gutierrez A., Segura-Carretero A. (2008). tabAnti-HER2 (erbB-2) oncogene effects of phenolic compounds directly isolated from commercial Extra- Virgin Olive Oil (EVOO). BMC Cancer.

[130] Menendez J.A., Vazquez-Martin A., Oliveras-Ferraros C., Garcia-Villalba R., Carrasco-Pancorbo A., Fernandez-Gutierrez A., Segura-Carretero A. (2008). Analyzing effects of extra-virgin olive oil polyphenols on breast cancer-associated fatty acid synthase protein expression using reverse-phase protein microarrays. Int. J. Mol. Med..

[131] Elamin M.H., Elmahi A.B., Daghestani M., Al-Olayan E.M., Al-Ajmi R.A., Alkhuriji A.F., Hamed S.S., El-Khadragy M.F. (2017). Synergistic Anti-Breast-Cancer Effects of Combined Treatment with Oleuropein and Doxorubicin In Vivo. Altern. Ther. Health Med..

[132] Liu L., Ahn K.S., Shanmugam M.K., Wang H., Shen H., Arfuso F., Chinnathambi A., Alharbi S.A., Chang Y., Sethi G. (2019). Oleuropein induces apoptosis via abrogating NF-κB activation cascade in estrogen receptor-negative breast cancer cells. J. Cell Biochem..

[133] Przychodzen P., Kuban-Jankowska A., Wyszkowska R., Barone G., Bosco G.L., Celso F.L., Kamm A., Daca A., Kostrzewa T., Gorska-Ponikowska M. (2019). PTP1B phosphatase as a novel target of oleuropein activity in MCF-7 breast cancer model. Toxicol. In Vitro.

[134] Abtin M., Alivand M.R., Khaniani M.S., Bastami M., Zaeifizadeh M., Derakhshan S.M. (2018). Simultaneous downregulation of miR-21 and miR-155 through oleuropein for breast cancer prevention and therapy. J. Cell. Biochem..

[135] Rigacci S., Miceli C., Nediani C., Berti A., Cascella R., Pantano D., Nardiello P., Luccarini I., Casamenti F., Stefania R. (2015). Oleuropein aglycone induces autophagy via the AMPK/mTOR signalling pathway: A mechanistic insight. Oncotarget.

[136] Kikuchi M., Mano N., Uehara Y., Machida K., Kikuchi M. (2011). Cytotoxic and EGFR tyrosine kinase inhibitory activities of aglycone derivatives obtained by enzymatic hydrolysis of oleoside-type secoiridoid glucosides, oleuropein and ligustroside. J. Nat. Med..

[137] Busnena B.A., Foudah A.I., Melancon T., El Sayed K.A. (2013). Olive secoiridoids and semisynthetic bioisostere analogues for the control of metastatic breast cancer. Bioorg. Med. Chem..

